# 7.Q. Workshop: What population health researchers’ need and how PHIRI federated research infrastructure can help

**DOI:** 10.1093/eurpub/ckac129.466

**Published:** 2022-10-25

**Authors:** Organised by: PHIRI, EUPHA-PHMR, Chair persons: Enrique Bernal-Delgado (Spain), Martin Thißen (Germany)

## Abstract

What population health researchers’ need? The population health research community has a vast experience in the reuse of data for health monitoring and surveillance or healthcare performance assessment. However, there is a gap in the extensive reuse of individual sensitive data, particularly when mobilising these data requires the linkage of multiple data sources curated in different sites. The gap is greater when it comes to using sensitive data in cross-national research. The usual arguments to explain the scarce extensive and continuous mobilisation of sensitive data are data privacy and safety issues, the difficulty to discover data sources of value, complex accessing rules, uneven data quality (particularly, non-harmonized data), and limited capacity (personnel and dedicated resources). In InfAct Joint Action, Information for Action, we demonstrated at a very small scale that mobilising individual sensitive data is possible, it is compliant with the legal and ethical requirements, and it yields the expected outputs. The instrument used for such an achievement was the design, implementation and deployment of a very small-scale federated infrastructure, where we could pilot all the legal, organisational, data quality and technological issues related to the mobilisation of individual sensitive data. (https://doi.org/10.1186/s13690-021-00731-z). Building on those achievements In PHIRI (see here https://www.phiri.eu/wp7) we are paving the way for a large-scale research infrastructure where multiple population health researchers with multiple research questions will need the mobilisation of multiple data sources from multiple sites across Europe. The PHIRI enhanced infrastructure will have to be prepared to provide a variety of services for data discovery, data access, data analysis and research outputs FAIR publication, while improving the capacity of population health researchers community in the use of advanced computing tools. In this workshop we will start describing the PHIRI federated research infrastructure achievements, the governance step-wise approach and the technological solutions provided. The workshop will discuss how an enhanced PHIRI could improve its services for the community of population health researchers; in particular improving the analytical capacity and the associated technological solutions. Finally, the workshop will touch ground on the future developments, in particular, the interaction of the PHIRI infrastructure with existen European-wide services providers, as EGI, and research infrastructures.

**Key messages:**

• In the domain of population health sciences, the reuse of individual sensitive data for research purposes is very limited.

• The PHIRI federated research infrastructure is paving the way for population health researchers to enhance their research when reusing individual sensitive data.

